# The effect of hypoxia and hyperoxia on the growth and metabolic rate of *Rhinella marina* tadpoles

**DOI:** 10.1242/jeb.250327

**Published:** 2025-09-30

**Authors:** Cameron B. Schofield, Craig R. White

**Affiliations:** ^1^ School of The Environment, The University of Queensland, St Lucia, QLD 4072, Australia; ^2^ School of Biological Sciences, Monash University, Clayton, VIC 3800, Australia

**Keywords:** Scaling, Metabolic rate, Gill-oxygen limitation theory, Surface area

## Abstract

The gill-oxygen limitation theory (GOLT) hypothesises that specific growth rate slows as water-breathing ectotherms increase in size because their two-dimensional respiratory surfaces cannot keep up with the growth of their three-dimensional bodies. Thus, a declining relative oxygen supply causes the slowing and ultimately the cessation of growth. Here, we tested this hypothesis by rearing tadpoles *Rhinella marina* at four levels of aquatic oxygen (4, 10, 21 and 40 kPa) and measuring their growth rate and resting metabolic rate. We found that growth rates are positively related to environmental oxygen earlier in development, in support of GOLT, but that the difference in size among treatments disappears as animals continue to grow. At the time when among-treatment differences in growth are large, animals reared in hypoxia have elevated metabolic rate. This difference in metabolic rate is hypothesised to arise as a result of osmoregulatory costs associated with gill hypertrophy in hypoxia. We conclude that growth trajectories in tadpoles are shaped by allocation trade-offs among energy-demanding processes, operating within resource availability and supply constraints imposed by the environment and the physical geometry of exchange and transport systems.

## INTRODUCTION

The gill-oxygen limitation theory (GOLT) hypothesises that the proximate cause of growth trajectories in water-breathing ectotherms is the dimensional tension between the area of the surface through which oxygen is taken up (the gills) and the volume of the body that this surface must supply with oxygen (Pauly, 1981, 1997, 2021). GOLT can be expressed as a differential equation (Pauly, 2021; Pauly and Lam, 2023):
(1)

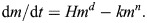
Eqn 1 describes the rate of change in mass *m* (growth rate, d*m*/d*t*) as the difference between two power functions, where *Hm^d^* and *km^n^* are conventionally considered to represent anabolic and catabolic processes (e.g. Pütter, 1920; Kearney, 2021) (*H* and *d* are the coefficient and exponent of the power function describing the relationship between anabolism and body mass, respectively, and *k* and *n* are the coefficient and exponent of the power function describing the relationship between catabolism and body mass, respectively). In GOLT, the anabolism term (*Hm^d^*) represents the rate of synthesis of body tissues fuelled by oxygen supplied across the surface of gills (Pauly, 2021). Gill surface area scales hypometrically with *d*<1 (Palzenberger and Pohla, 1992; Bigman et al., 2018; Scheuffele et al., 2021; Wegner and Farrell, 2024), and so the anabolism term is also expected to scale hypoallometrically as a consequence of this geometric constraint (Pauly, 2021). The catabolism term (*km^n^*) is interpreted by GOLT to represent the spontaneous denaturation of the proteins and other molecules that contribute to body mass, the rate of which is assumed to be directly proportional to body mass (i.e. *n*=1) (Pauly, 2021; Pauly and Lam, 2023). Growth ceases at maximum size when *Hm^d^*=*km^n^*.

If *n*>*d*, Eqn 1 suggests that the rate of growth is increasingly constrained as animals increase in size, as the rate of catabolism converges on the (increasingly oxygen constrained) rate of anabolism. Assuming *n*=1, Eqn 1 can be expressed as specific growth rate:
(2)

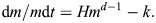
Thus, assuming that the mass-specific rate of anabolism (*k*) is independent of ambient oxygen, specific growth rate is expected to decline monotonically with size. Oxygen supplementation should relieve this constraint at all values of *m*, but most substantially at higher *m*.

GOLT can therefore be tested by manipulating environmental oxygen availability and determining the size at which animals reach maturity (Kolding et al., 2008; Pauly, 2021), as well as the rate at which they grow. GOLT would be supported if size at maturity, asymptotic size and growth rate at a given size increase with environmental oxygen. Such tests should ideally be conducted within species, as among-species patterns might obscure size-related within-species (i.e. ontogenetic) effects (Müller et al., 2023).

Toads of the family Bufonidae provide a suitable experimental system in which GOLT can be tested, because bufonid tadpoles do not have functional lungs until immediately before the completion of metamorphosis, and therefore do not breathe air (Savage, 1952; Wassersug and Seibert, 1975; Feder, 1983a; Phillips et al., 2024). We therefore tested GOLT by exposing cane toad, *Rhinella marina*, tadpoles to aquatic hypoxia or hyperoxia and exploring the effects of this on growth and metabolic rate.

## MATERIALS AND METHODS

### Animal collection and maintenance

All experiments were approved by the University of Queensland NEWMA Animal Ethics Committee. Multiple clutches of *Rhinella marina* (Linnaeus 1758) eggs were collected simultaneously from submerged vegetation within a lake in the Brisbane Botanical Gardens, Mt Coot-tha Brisbane, QLD, Australia. The egg strings were then transported to The University of Queensland in plastic containers filled with sufficient lake water for all ova to remain submerged.

**Fig. 1.**
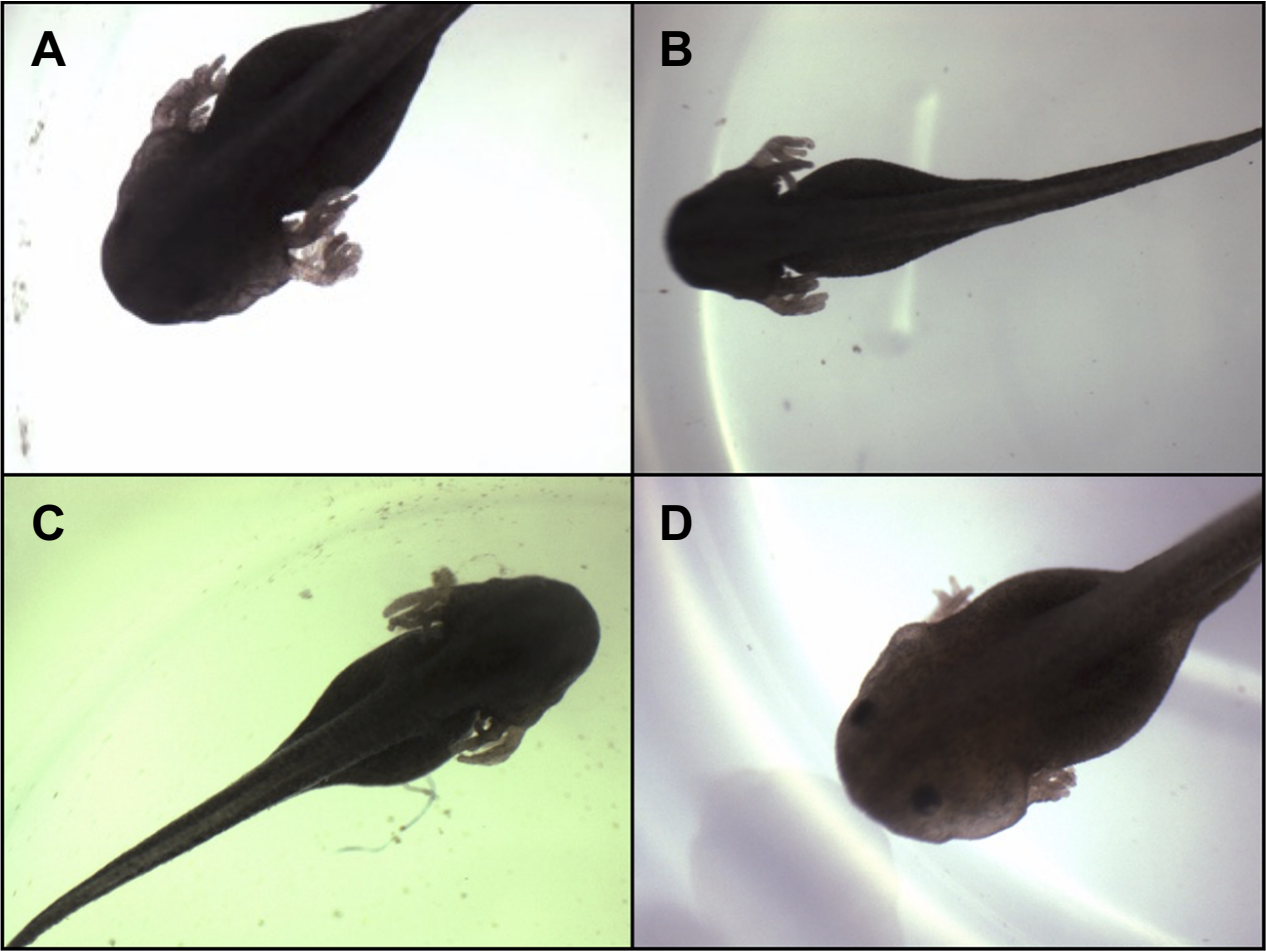
**Example images of *Rhinella marina* larvae showing modified gill morphologies due to chronic exposure to varied *P*_O_2__.** Oxygen availability values for the acclimating larvae were 4 kPa O_2_ (A), 10 kPa O_2_ (B), 21 kPa O_2_ (C) and 40 kPa O_2_ (D). All images were taken from larvae at approximately Gosner stage 22 of development, and prepared using a dissecting microscope with a top-mounted digital camera.

Upon arrival at the laboratory, egg clutches were mixed before being separated and randomly allocated and transferred to four identical acclimation rearing containers filled with 22 l of deionised (reverse-osmosis) water mixed with aquarium salts (∼3 g l^−1^) for essential ions. Acclimation containers were made of dark-coloured plastic with tight-fitting translucent white lids to allow light into the tubs and were positioned randomly within the room. The four treatment groups differed only in the dissolved oxygen content of the water, consisting of severely hypoxic, hypoxic, normoxic and hyperoxic treatments with oxygen partial pressures (*P*_O_2__) of 4, 10, 21 and 40 kPa (±1 kPa), respectively. Levels of dissolved oxygen were maintained by bubbling commercially prepared gas mixtures of O_2_ in N_2_ (BOC Gases, Brisbane, QLD, Australia) (Warkentin, 2002; Schimpf et al., 2009) into the middle of the water in each container, thus ensuring that the water was well mixed and that *P*_O_2__ in the air space above the water was matched with that of the water itself. The manipulation of oxygen was sufficient to generate qualitative differences in gill morphology (Fig. 1), as has been observed in other species exposed to environmental hypoxia (e.g. Burggren and Mwalukoma, 1983). Larvae were provided with an *ad libitum* diet of pre-boiled spinach. Partial water changes (⅓ total volume) were carried out twice weekly and spinach was replaced at least every second day. Eggs and larvae were housed in a controlled-temperature room at 25°C (±1°C) and a 12 h:12 h photoperiod centred on 12:30 h.

### Respirometry

Acclimated larvae were randomly selected and transferred to sealed vials for measurement of aquatic oxygen consumption using optical fluorescence-based closed system respirometry (Koster et al., 2008). Oxygen consumption rates were frequently recorded throughout the entire developmental period. The rate of oxygen consumption (*V̇*_O_2__) was measured during the photophase of the larvae's photoperiod as this is their less active period (Freeland and Kerin, 1991; Hearnden, 2006; Smith et al., 2007). Twenty-four hours prior to *V̇*_O_2__ measurement, selected larvae were transferred to plastic-framed mesh cages within the acclimation enclosures to limit their access to food. However, anuran larvae are known to consume most forms of detritus including their own faecal material (Hoff et al., 1999), as well as possessing yolk reserves up to Gosner stage 22 (Gosner, 1960; Thibaudeau and Altig, 1999); hence, they were unlikely to be post-absorptive. The aquatic *V̇*_O_2__ of each larva was measured in individual vials containing a planar oxygen-sensitive spot mounted on the bottom internal surface (W-In-SP-PSt3-NAU-D5-YOP, PreSens, Regensburg, Germany). The chambers were selected to be sufficiently large that the movement of larvae was not restricted. Small larvae were measured in chambers with a volume of 5 ml while larvae greater than ∼200 mg were measured in 25 ml chambers. To eliminate any confounding factors between measurement and acclimation conditions, all larvae were measured in air-saturated deionised water mixed with aquarium salts at 25°C. We chose this approach so that we could be confident that any effect of rearing oxygen level on *V̇*_O_2__ could be unambiguously attributed to rearing conditions, rather than to the level of oxygen that the animals were exposed to during the measurement of *V̇*_O_2__. We note, however, that future work could explore this issue in more detail by undertaking respirometry measurements of animals from each treatment condition at all treatment levels of O_2_ if measurement capacity allows (i.e. animals exposed to 4 kPa O_2_ during development could be measured for *V̇*_O_2__ at each of 4, 10, 21 and 40 kPa O_2_). Measurements at this scale were, unfortunately, not possible in the present study.

Developing anuran larvae are known to utilize aerial respiration at the water surface, known as air-gulping (Bradford, 1983; Feder, 1983b; Feder and Wassersug, 1984). Surfacing was rarely observed throughout development in the present study, suggesting that aerial respiration is minimal in *R. marina* tadpoles, and bufonid tadpoles do not breath air (Savage, 1952; Wassersug and Seibert, 1975; Feder, 1983a; Phillips et al., 2024). Thus, *V̇*_O_2__ of tadpoles was measured solely as aquatic respiration. Once larvae were individually sealed within the chambers, the oxygen content of the water was monitored using a SensorDish Reader (PreSens) (Koster et al., 2008). Vials containing only water identical to that used for each larval chamber were also measured at the same time to quantify microbial oxygen consumption. Temperature was monitored throughout measurements and all vials were positioned on a variable speed, orbital platform mixer (Ratek Instruments, Boronia, VIC, Australia) that gently stirred water within the vials and ensured adequate mixing. All measurements occurred within a darkened section of the controlled-temperature room at 25°C (±1°C) to ensure minimal stress and activity. Oxygen content was recorded at 2 min intervals and larvae were contained within the vials until oxygen concentration had fallen by a minimum of 10% air saturation.

Following oxygen consumption measurements, each larva's wet body mass was weighed to the nearest 0.1 mg using a mesh weighing stage to enable removal of all excess water. The Gosner developmental stage (Gosner, 1960) of each larva was then noted using a dissecting microscope with mounted digital camera. Larvae were then euthanised by overdose of MS-222 (Sigma-Aldrich, St Louis, MO, USA).

### Metabolic rate calculation

Rates of oxygen consumption were calculated as millilitres of O_2_ consumed per hour, by fitting a linear regression to the data of oxygen content over time to calculate the rate of oxygen consumption:
(3)

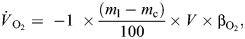
where *m*_l_ is the rate of change of oxygen saturation within a chamber containing a larva (% air-saturation h^−1^), *m*_c_ is the average slope calculated for all control chambers included in the measurement period (% air-saturation h^−1^), *V* is the volume of water in the respirometry chamber (=0.005 l or 0.025 l minus the volume of larva) and β_O_2__ is the oxygen capacitance of air-saturated freshwater at 25°C (5.77 ml l^−1^) (Riley and Chester, 1971). Rates of aquatic oxygen consumption (ml O_2_ h^−1^) were converted to metabolic rate (J min^−1^) assuming a respiratory quotient of 0.8 and an oxyjoule equivalent of 20.5 J ml^−1^ (Koteja, 1996; Lighton, 2008).

### Statistical analysis

Data for age, mass and metabolic rate were log_10_-transformed prior to analysis. Oxygen treatment was treated as a categorical fixed effect. Data for growth were analysed using a linear model with log_10_(mass) as a response and log_10_(age), [log_10_(age)]^2^, treatment and the interaction between log_10_(mass) and treatment and between [log_10_(age)]^2^ and treatment, followed by ANOVA with Type III sums of squares. Data for metabolic rate were analysed with log_10_(metabolic rate) as a response and log_10_(mass), treatment and their interaction as fixed effects, followed by ANOVA with Type III sums of squares.

## RESULTS

**Fig. 2.**
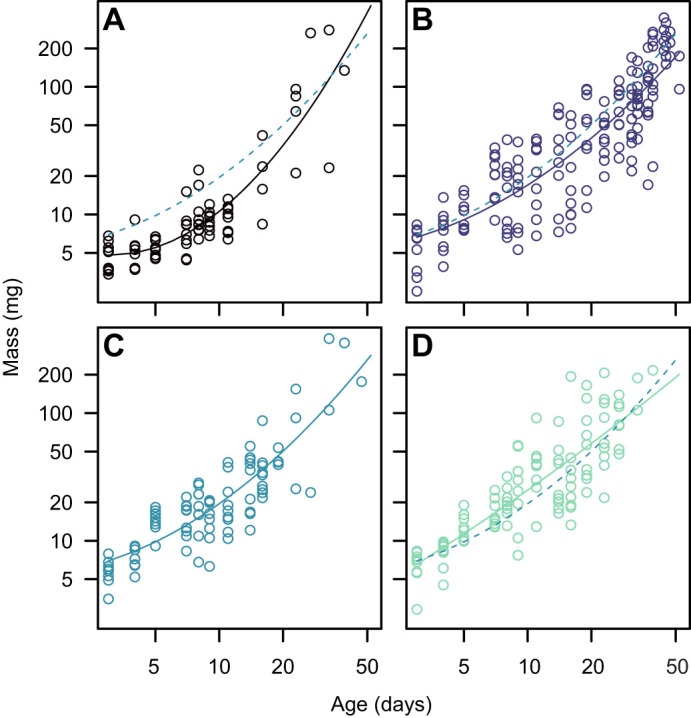
**The effect of environmental oxygen on growth in *R. marina* tadpoles.** Data are for larvae acclimated to 4 kPa O_2_ (A), 10 kPa O_2_ (B), 21 kPa O_2_ (C) and 40 kPa O_2_ (D). The dashed lines in A, B and D represent the fit to data for animals reared in normoxia (C).

**Fig. 3.**
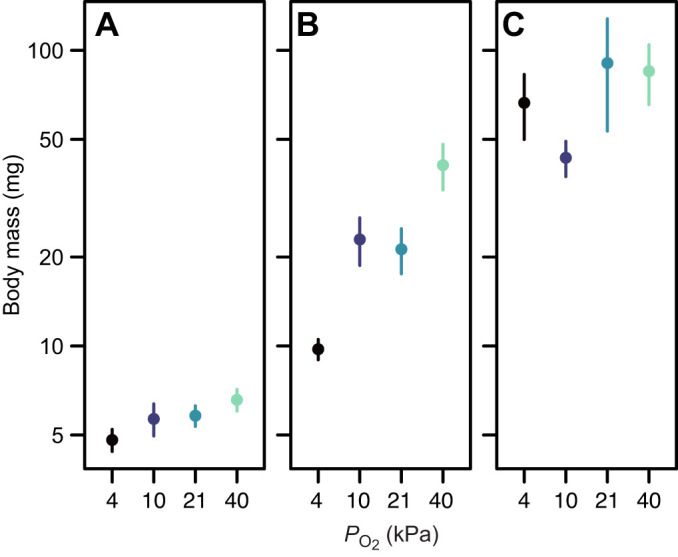
**The effect of environmental oxygen on body mass at different ages in *R. marina* tadpoles.** Mass (means±s.e.m.) was measured at 3 days (A), 11 days (B) and 23 days post-hatch (C).

The relationship between mass and age was well described by a polynomial in which the coefficients associated with both the linear and quadratic terms were affected by treatment: treatment× log_10_(age): *F*_3,430_=2.98, *P*=0.03; treatment×[log_10_(age)]^2^: *F*_3,430_= 3.34, *P*=0.02 (Fig. 2). The most substantial differences among treatments were observed in the middle of development, around day 11, and were largely absent by day 23 (Fig. 3).

Treatment had a significant effect on the scaling exponent (*b*) of metabolic rate [treatment×log_10_(mass): *F*_3,405_=5.07, *P*=0.002]. The scaling exponent of metabolic rate was steepest at 10 kPa oxygen [*b*_10_=0.86±0.03 (s.e.e.)] and shallower in severe hypoxia (*b*_4_=0.74±0.05), normoxia (*b*_21_=0.77±0.06) and hyperoxia (*b*_40_=0.66±0.06).

**Fig. 4.**
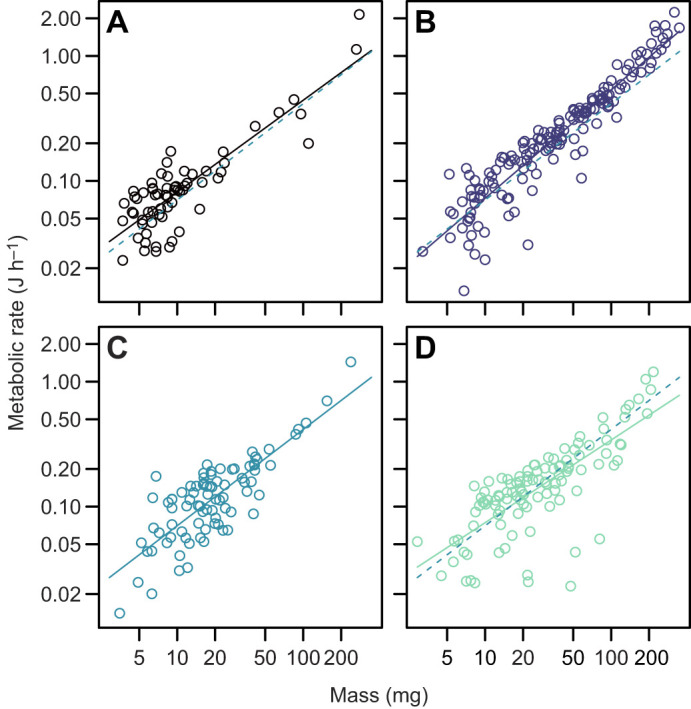
**The effect of environmental oxygen on the scaling of metabolic rate in *R. marina* tadpoles.** Data are for larvae acclimated to 4 kPa O_2_ (A), 10 kPa O_2_ (B), 21 kPa O_2_ (C) and 40 kPa O_2_ (D). The dashed lines in A, B and D represent the fit to data for animals reared in normoxia (C).

**Fig. 5.**
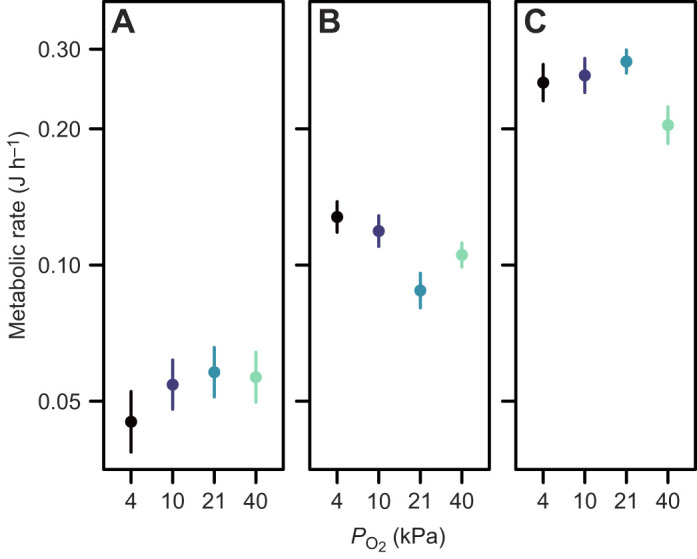
**The effect of environmental oxygen on metabolic rate at different ages in *R. marina* tadpoles.** Metabolic rate (means±s.e.m.) was measured at 3–5 days (A), 8–11 days (B) and 19–23 days post-hatch (C). Within each panel, data were normalised to a common mass (A: 7.8 mg, B: 16.6 mg, C: 52.7 mg) using the common parameter estimate for the effect of log_10_(mass) on log_10_(metabolic rate) for the indicated age range, from a linear model with treatment (4, 10, 21 or 40 kPa) as a fixed effect.

For metabolic rate across days 8–11, a period leading into the time at which growth differences were substantial (Fig. 3B), the effect of mass on metabolic rate was consistent among treatments [treatment×log_10_(mass): *F*_3,96_=0.64 *P*=0.59] and there was a significant effect of treatment on metabolic rate in an ANCOVA model in which there was no treatment×log_10_(mass) interaction (*F*_3,99_=4.13, *P*=0.008) (Fig. 4). The pattern of variation was that metabolic rate was elevated when tadpoles were reared at 4% oxygen (*t*_99_=3.16, *P*=0.002) and 10% oxygen (*t*_99_=2.77, *P*=0.007), but not 40% oxygen (*t*_99_=1.60, *P*=0.11) (Fig. 5B).

Metabolic rate did not differ among treatments at ages 3–5 days (ANCOVA, *F*_3,83_=0.09, *P*=0.96; Fig. 5A), if we ignored a significant treatment×log_10_(mass) interaction (*F*_3,80_=3.41, *P*=0.02; this interaction was driven by a single large individual reared at 4 kPa that had a large mass and low metabolic rate – the interaction was no longer significant if this individual was removed: *F*_3,79_=1.92, *P*=0.13).

For metabolic rate at ages 19–23 days, a period leading into the time at which mass became relatively uniform (Fig. 3C), there was no significant treatment×log_10_(mass) interaction (*F*_3,38_=1.57, *P*=0.21) and metabolic rate did not differ significantly among treatments (ANCOVA *F*_3,41_=2.00, *P*=0.13; Fig. 5C).

## DISCUSSION

The major finding of the present study is that growth rate is substantially higher in cane toad tadpoles reared in elevated oxygen (Fig. 2). This generates dramatic differences in mass during the earlier part of development (Fig. 2A,B) but these differences do not persist later in development (Fig. 2C). These findings provide support for GOLT, because growth rate is positively related to aquatic oxygen availability. It is interesting, however, that the observed differences in growth rate do not alter size later in development, where GOLT predicts that oxygen limitation will be most severe. It is possible that the qualitative differences in gill morphology that we observed (Fig. 1) manifest only later in development, removing or alleviating the effect of environmental oxygen, but quantitative evaluation of gill morphology throughout development (and, ideally, perfusion) would be necessary to explore this in detail. Nonetheless, it is clear that tadpoles in extreme hypoxia exhibit rapid growth late in development (Fig. 2A), which is likely facilitated by increased oxygen supply through increased gill surface area (Fig. 1A).

McArley et al. (2021) undertook a qualitative meta-analysis of the results of 30 studies of fish that measured growth performance in response to hyperoxia. Only seven of these studies reported significantly improved growth performance in hyperoxia; 20 reported no effect of hyperoxia, and four reported significantly impaired growth in hyperoxia. Studies of air-breathing terrestrial animals have also been mixed: hyperoxic exposure increases growth or development rates of some species, including eastern fence lizards (*Sceloporus undulatus*) (Andrews, 2002), American alligator (*Alligator mississippiensis*) (Owerkowicz et al., 2009) and Mongolian racerunner lizards (*Eremias argus*) (Sun et al., 2014), but not northern bobwhite (*Colinus virginianus*) (Williams and Swift, 1988). For cockroaches (*Nauphoeta cinerea*), the effect of hyperoxia on growth is positive at 25°C (Bartrim et al., 2014), but absent at 28°C and negative at higher temperatures (Lombardi et al., 2020). Hyperoxia reduces growth rate in *Drosophila melanogaster* (Farzin et al., 2014) and the effect of hyperoxia on growth rate in mealworms *Zophbas morio* depends on rearing density (VandenBrooks et al., 2020). This body of work suggests that oxygen limitation of growth is not ubiquitous and not limited to aquatic animals. A valuable next step would be to undertake a quantitative synthesis and meta-analysis of the literature (Gurevitch et al., 2018; O'Dea et al., 2021; Nakagawa et al., 2023), to obtain reliable evidence of the effect of oxygen – particularly hyperoxia – on growth (noting that elevated oxygen can also act as a stressor: McArley et al., 2021; Müller and Pauly, 2024).

### Why do tadpoles grow slower in hypoxia?

Tadpoles reared in hyperoxia in the present study showed no change in resting metabolic rate when measured in normoxia. Thus, there is no evidence that tadpoles grow faster in hyperoxia because of increased energy allocation of tissue biosynthesis (Wieser, 1994; Rosenfeld et al., 2015), facilitated by elevated environmental oxygen. Yet metabolic rate was elevated for animals reared in hypoxia around the time at which growth trajectories diverged most dramatically (Figs 3B and 5B). This suggests that hypoxia presents oxygen challenges that slow early growth, which are overcome only through metabolic investment. Thus, the elevation in metabolic rate could arise because of increased investment in gills in hypoxia (Fig. 1), which requires investment in tissue synthesis and incurs osmoregulatory costs associated with increased gill area, and trades off against energy allocation to growth. Arguing against the increased osmoregulatory cost hypothesis, gills account for a relatively small portion of resting metabolic rate in fish (e.g. <5% in cutthroat trout *Oncorhynchus clarki clarki*: Morgan and Iwama, 1999), and hypoxia-induced gill remodelling in fish does not appear to compromise osmoregulation because of reductions in gill ionic permeability during hypoxia (reviewed by Gilmour and Perry, 2018). An alternative possibility is that the increase in environmental oxygen availability associated with the transition from hypoxia to normoxia for respirometry measurements allowed animals to invest in metabolic activities that were previously restricted by hypoxia, such as biosynthesis and self-maintenance. Measurements of animals in a factorial design including all combinations of acclimation and measurement oxygen levels would be valuable to disentangle these possibilities. Such measurements would make it possible to determine whether the relationship between environmental oxygen and growth rate in *R. marina* tadpoles arises because of a limitation of environmental oxygen, because of elevated maintenance costs, or both.
